# A New Approach to Accuracy Evaluation of Single-Tooth Abutment Using Two-Dimensional Analysis in Two Intraoral Scanners

**DOI:** 10.3390/ijerph16061021

**Published:** 2019-03-20

**Authors:** Jiyoun Maeng, Young-Jun Lim, Bongju Kim, Myung-Joo Kim, Ho-Beom Kwon

**Affiliations:** 1 Department of Dentistry, School of Dentistry, Seoul National University, Seoul 03080, Korea; jymaeng@snu.ac.kr; 2 Department of Prosthodontics and Dental Research Institute, School of Dentistry, Seoul National University, Seoul 03080, Korea; silk1@snu.ac.kr (M.-J.K.); proskwon@snu.ac.kr (H.-B.K.); 3 Clinical Translational Research Center for Dental Science, Seoul National University Dental Hospital, Seoul 03080, Korea; bjkim016@gmail.com

**Keywords:** intraoral scanners, accuracy, 2-dimensional analysis, internal deviation, external deviation, RMS

## Abstract

The aim of this study was to two-dimensionally evaluate deviation errors at five digital cross-sections of single-tooth abutment in regards to data obtained from two intraoral scanners, and to evaluate accuracy of individual scanners. Two intraoral scanners, the Trios 3^®^ (3 Shape, Copenhagen, Denmark) and EzScan^®^ (Vatech, Hwaseong, Korea), were evaluated by utilizing 13 stone models. The superimposed 3D data files were sectioned into five different planes: buccal-lingual section (BL), mesial-distal section (MD), transverse high section (TH), transverse middle section (TM), and transverse low section (TL). Accuracy comparison between the two scanners in 5 groups was performed. BL vs. MD of each scanner, and three transverse groups (TH, TM, TL) of each scanner were analyzed for accuracy comparison. In comparison of 2-D analyses for two intraoral scanners, Trios 3^®^ showed statistically significant higher accuracy in root mean square (RMS) at BL, TH, and TL (*p* < 0.05). For each scanner, RMS value showed that mesial-distal sections were more prone to error than buccal-lingual section, which exhibited statistically significant errors (*p* < 0.05) while the transverse groups did not. Two-dimensional analysis is more insightful than three-dimensional analysis on single-tooth abutment. In mesiodistal areas, rough prepped areas, and sharp edges where scanner accessibility is difficult, high deviation errors are shown.

## 1. Introduction

Digitalization is no longer an unfamiliar concept; application of digitization is trending in social as well as the dental community. There have been continuous movements to shift the paradigm to digital dentistry, and computer-assisted technologies have constantly evolved to meet the needs.

Although there is no doubt in the comfort that complete digital flow can provide to patients as well as clinicians and technicians, there are still doubts to whether digital technology such as intraoral scanners are sufficiently reliable enough to be applied to daily clinical environment. Even now, continuous efforts are made to assess and verify the accuracy and clinical applicability of existing and newly introduced intraoral scanners. However, there is no universal consensus. Clinicians and technicians are hence left to choose between digital and conventional procession [1,2,3].

In prosthetic rehabilitation of single tooth, accuracy of abutment is important. Therefore, many previous and ongoing studies continue to test accuracy of intraoral scanners [3,4,5]. In a recent study on accuracy of digital dental scanners, the author compared digital and conventional methods with teeth of nine different convergence angles. Three-dimensional analysis was performed for single-abutment tooth. RMS (root mean square) values revealed that abutment tooth geometry influenced conventional methods and model scanners, but intraoral scanners showed consistent accuracy regardless [6]. Another study evaluated trueness and precision of six intraoral impression systems with varying scan techniques. Best-fit algorithm values achieved from three-dimensional superimposition of obtained virtual models revealed scan patterns did not significantly influence the accuracy of digital impression systems [7]. As such, most accuracy studies that concluded that current intraoral scanners perform with similar reliability used three-dimensional analysis. There is a lack of studies utilizing two-dimensional analysis on accuracy of intraoral scanners.

Most 3-dimensional comparative analyses use data of abutment tooth and its surrounding soft tissue for best-fit algorithm. Because the collected data includes adjacent anatomy, which is not the actual target of investigation, there is increase in data gathering [8]. Best-fit algorithm with gathered data results in a mean value of both the abutment tooth and its surrounding tissue. Unfortunately, actual information that we need is the accuracy of abutment, not that of surrounding soft tissue. Increase in unnecessary data results in undesired correction of errors, and the tendency to modify actual errors makes it hard to identify accuracy deviations exclusively of a single tooth abutment. 

Although surrounding anatomy is not excluded in scanned data, three-dimensional analyses do not include a process to compensate for this factor of fallacy. Because surrounding anatomy is included in scanned data, succeeding analysis will likely also include increased deviations or compensations arising from surrounding soft tissues. Therefore, in three-dimensional evaluation, true errors solely from prepared tooth cannot be measured. 

Three-dimensional analysis provides insufficient evidence to address existing errors of scanners. In fact, inability to point out inaccuracies often results in study conclusions that are rebated under clinical settings. For this reason, it seemed reasonable and necessary to come up with a 2-dimensional analysis method as a means to assess accuracy of intraoral scanners. Therefore, the aim of this study was to evaluate accuracy outcomes of two types of scanners through 2-dimensional analyses at five digital cross-sections (mesiodistal, buccolingual, high transverse, mid-transverse, low transverse) of single-tooth abutment, and to evaluate accuracy of individual scanners. 

## 2. Materials and Methods 

This study utilized improved stone cast models of 13 participants of clinical study (IRB No. ERI 18017) performed in Seoul National University Dental Hospital (SNUDH). Casts for this study were fabricated via conventional impression method. The study sample consisted of six males and seven females with an average age of 53.92 (±10.43). One tooth per study participant was selected for single-tooth abutment model: one upper 1st premolar, three upper 2nd premolar, four upper 1st molar and five lower 1st molar teeth (total number of teeth: 13). 

All teeth were prepared as follows. For occlusal reduction, 1.5 mm reduction in functional cusp and 1.0 mm reduction in nonfunctional cusp were made, in addition to 1.0–1.2 mm circumferential reduction. All axial walls of the preparations were tapered to 6–10°. Margins were set 0.5–1.0 mm supra-gingivally, with distinct shoulder finish line (>0.8 mm). All edges were rounded. 

Two types of intraoral scanner systems were tested for this study: Trios 3^®^ (3 Shape, Copenhagen, Denmark) and EzScan^®^ (Vatech, Hwaseong, Korea). Identica Hybrid^®^ (Medit Co, Seoul, Korea) was used to create digital reference data of 13 models.

**Figure 1:**
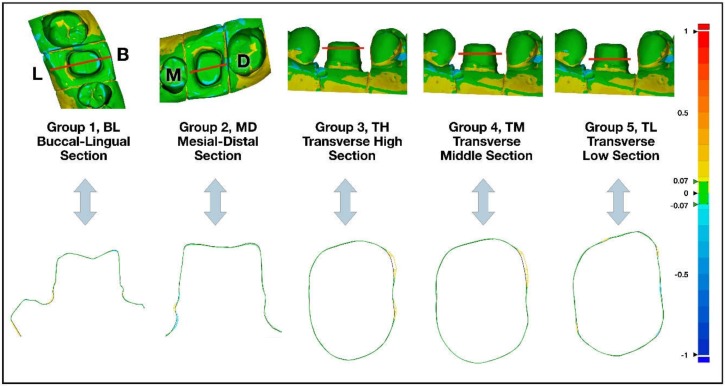
Superimposed 3D data images were sectioned to five following 2D planes: buccal-lingual section (BL, group 1), mesial-distal section (MD, group 2), transverse high section (TH, group 3), transverse middle section (TM, group 4), and transverse low section (TL, group 5).

Scan files of the two experimental scanners were exported to Geomagic Control X^TM^ (3D systems, RockHill, SC, USA). Using Best-Fit Alignment function in Geomagic Control X, acquired data and reference data were 3-dimensionally aligned. The alignment was set to produce minimal error based on least square regression. Superimposed 3D data files were then cut to five cross-sectional planes: buccal-lingual section (BL, group 1), mesial-distal section (MD, group 2), transverse high section (TH, group 3), transverse middle section (TM, group 4), and transverse low section (TL, group 5). The five cross-sectional plans used in the analysis are shown in detail in Figure 1.

The 2D Compare function of Geomagic Control X was used to analyze discrepancies of superimposed cross-sections, with a set tolerance of ±0.07 mm and a maximum tolerance range of ±1.0 mm. For all comparisons, discrepancies between experimental file and reference file were expressed with a + or − sign, indicating deviation directionality (internal; −, external; +). These values were also visually expressed with a range of color-codes, where yellow lines indicate external deviations and cyan lines indicate internal deviations. The deviation (internal; −, external; +) and root mean square (RMS) were numerically quantified with a mean and standard deviation, respectively. To ensure consistency, single proficient investigator was responsible for performing all measurements with two intraoral scanners. The acquired two-dimensional data were re-measured by the same investigator as an intra-examiner test.

### Statistical Analysis

Statistical analysis was carried out with SigmaPlot^TM^ (Systat Software Inc., San Jose, CA, USA). Comparison between the two scanners regarding accuracy in 5 cross-sectional groups was performed using paired *t*-test. For each of the scanners, *t*-test was used for comparison of BL (group 1) and MD (group 2). One-way ANOVA test was used for comparison of TH (group 3), TM (group 4), and TL (group 5). Fisher’s Least Significant Difference method was used for post-hoc test. Mean and standard deviation were calculated based on data with a significance level of 0.05. All variables were presented with a mean and a standard deviation, where *p* < 0.05 was considered statistically significant.

## 3. Results

**Figure 2:**
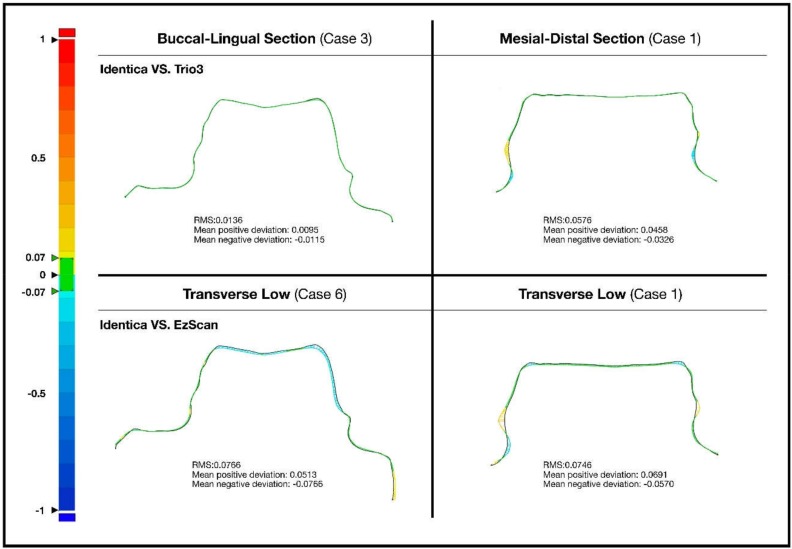
Comparison of the bucco-lingual section and mesial-distal section in cases with pronounced error.

**Figure 3:**
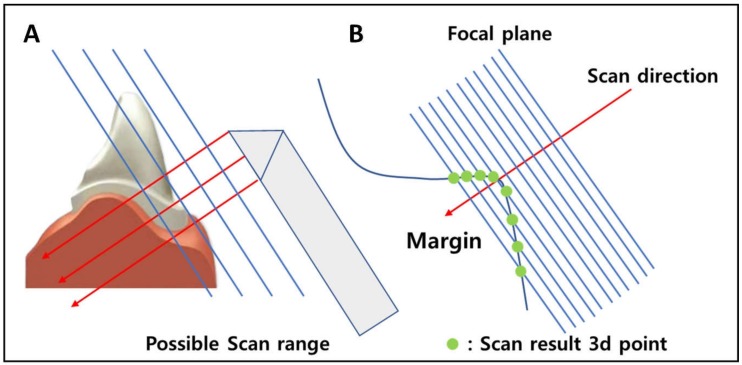
(**A**) Optimal scan data is acquired when scanner is positioned at the optimized focal-depth of scanner. Distance between target point and scanner is greater in mesial-distal area compared to that in buccal-lingual area, causing deviation errors in scanning. (**B**) Optimal scan data is acquired when scan direction is perpendicular to the focal plane. Appropriate angular approach is easily accessible for buccal-lingual margin, but not for mesial-distal margin due to anatomical hindrance.

**Figure 4:**
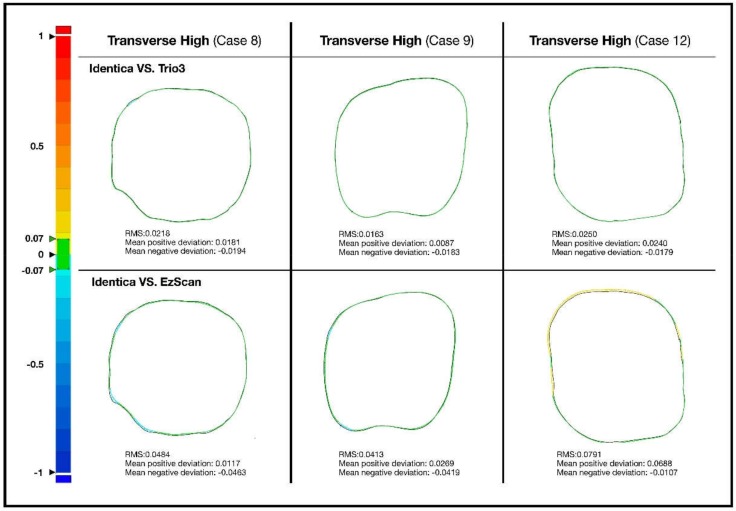
Comparison of transverse high section, transverse middle, and transverse low section in extracted study cases with pronounced error.

Accuracy of the two scanners against model scanner was measured from 5 different cross-sections. Color-coded image data of two-dimensional analysis on accuracy of Trios 3 and EzScan against model scanner can be seen in Figure 1, Figure 2, Figure 3 and Figure 4. Differences of 13 study casts were numerically displayed with RMS, mean positive deviation, and mean negative deviation values. The results of 2-dimensional analysis for two intraoral scanners at 5 cross sections were listed in Table 1. 

Comparison of accuracy of the two scanners displayed statistically meaningful differences in group 1 (buccal-lingual), 3 (transverse-high), and 5 (transverse-low). At buccal-lingual section, Trios 3 was more accurate than EzScan. At mesial-distal section, both Trios 3 and EzScan exhibited high deviations. RMS and mean deviation values of groups 2 (mesial-distal) exhibited no statistically meaningful difference between Trios 3 and EzScan. No significant differences in group 2 may be interpreted as scanner’s inability to access certain areas, or with depth made scanned images more susceptible to imprecisions. RMS and mean negative deviation of group 4 (transverse-middle) values were not statistically significant. It can be speculated that both scanners reproduce better scan images in smooth surfaces and poor images in irregular segments (edge, margin). 

For the second part of the study, accuracy at different cross-sections was compared for each scanner. Buccal-lingual (group 1) vs. mesial-distal (group 2) of each scanner, and three transverse groups (group 3, 4, 5) of each scanner were analyzed for accuracy comparison. The results of 2-dimensional analysis for accuracy at BL vs. MD and TH vs. TM vs. TL for each intraoral scanner expressed with RMS, mean positive deviation, and mean negative deviation values were listed in Table 2.

In comparison between the vertical cross sections, RMS and mean negative deviation values exhibited statistically significant difference while mean positive deviation value did not. Comparison of the transverse groups did not exhibit statistical significance. Accuracy comparison of group 1 and group 2 for each scanner showed that mesial-distal sections are more prone to error than buccal-lingual section, and statistically significant errors are expressed as negative deviations for both scanners (*p* = 0.028, 0.034). Comparatively, Trios 3 was superior in reproducing irregular surfaces in single-tooth abutment scan.

## 4. Discussion

Studies assessing the accuracy of intraoral scanners via 2-dimensional analysis method are scarce. Most 3-dimensional analysis methods collect data of target tooth as well as surrounding soft tissue. However, this increase in data can distort accuracy of digital dental impression. Previous studies have found lower accuracy when quadrant was scanned instead of single tooth [9]. Increase in gathered data results in falsely compensated values of best-fit algorithm. Therefore, with three-dimensional analysis, evaluation of accuracy solely on tooth is difficult. In fact, 3D analysis method’s failure to detect inaccuracies often lead to confirmation of digital scanners as reliable alternative to conventional impression methods. For the reason, we came up with a 2-dimensional analysis method to more precisely assess deviations of the scanners. 

Both the Trios 3^®^ and EzScan^®^ systems featured common weakness but at different levels. Within the finite boundaries of this study, Trios 3 was generally better at generating an accurate digital copy than EzScan. In buccal-lingual cross-section, Trios 3 showed statistically significant superiority, indicating Trios 3 performs with better accuracy on surfaces that are easily accessible. In mesial-distal cross-section, both scanners performed poorly. There was no significant difference between the two scanners, which could be interpreted as intraoral dental scanner’s inherent imperfection to obtain data in deep and narrow areas (Figure 2). 

All scanners presented in current market have their own optimized focal-depth. It is important that scanner is located exactly at this optimized location, not further or closer, in order to acquire appropriate scan data. Having a focal-depth range, thereby, makes data obtainment easier since small room for freedom is given to where scanner can be located for optimal scan. Therefore, control of distance between the target and scanner is important. In Trios3, buccal-lingual data showed bigger deviation error than those of mesial-distal data. Such discrepancy could be explained by anatomical differences of buccal-lingual area and mesial-distal area. Depth control is easier bucco-lingually, but depth control is harder mesio-distally especially in regions where neighboring teeth or maxillofacial anatomical features exist. Because access itself is comparatively difficult in mesial-distal area, placing the scanner at optimal focal-depth is also hard. 

Moreover, scanners show superior data obtaining performance when scan direction is perpendicular to focal plane, which is also difficult in mesial-distal areas due to anatomical hindrances such as neighboring teeth. due to accessibility problems which leads to scan direction problems, mesial-distal margins cannot create significant angular differences which allows optimal data acquisition. The same line of logic would be applicable to errors featured by EzScan (Figure 3).

Even within transverse cross-sections, different levels of deviations were observed. In the highest and lowest transverse cross-sections, both intraoral scanners performed with fluctuating accuracies, while no significant imprecisions were observed at mid-transverse cross-section. Trios 3 demonstrated comparatively less errors than EzScan, nevertheless (Figure 4).

The present study was designed not only to compare the reliability of the intraoral scanners but also to determine error-susceptible surfaces of each scanners. Accuracy measurements of the two scanners revealed that Trios 3 executed more accurately than EzScan in comparatively accessible sections such as buccal and lingual surfaces. 

**Figure 5:**
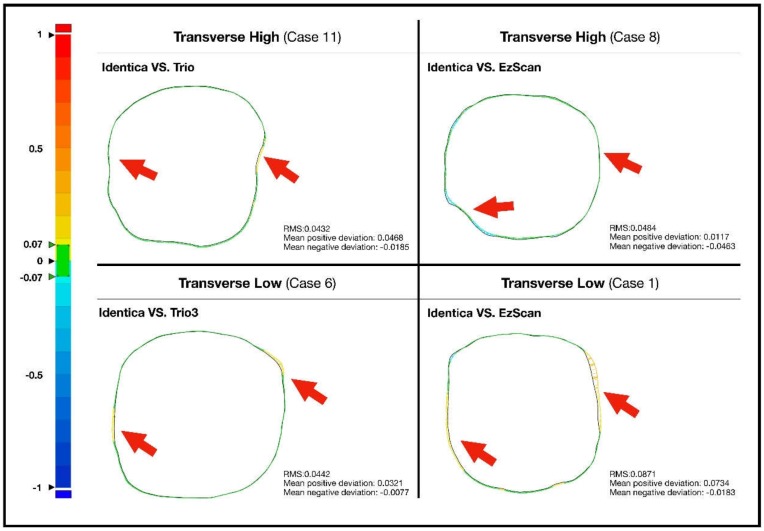
Sharp edges and rough areas on prepped surfaces showed high deviation errors. Red arrows indicate pronounced errors.

In proximal areas where access is harder, however, comparative deviations were commonly observed in both intraoral scan systems. Increase of deviations was noticeable in both scanners especially where bumps or sharp angles existed. This consistency could also be found in transverse sections; sharp or roughly prepped surfaces from coronal views revealed higher deviation for both scanners. Steep axial walls showed consistent deviations in all transverse cross-sections (Figure 5).

Results and analysis of present study lead to the common conclusion that intraoral scanner performance is highly influenced by (1) the type of scanner used and (2) structural components of the tooth involved. Similar conclusions have been derived in a number of studies regarding the importance of tooth geometry. 

Yang et al. [10] evaluated digital impressions of single crown attained with three digital scanner systems. Margins and distal surfaces showed greater deviations, especially in narrow or angular areas such as interproximal surfaces. The study reported that fallacies of digital impression increase when prominent angle bigger than 60° exist between scanner and the perpendicular of target surface. DeLong et al. [11] also reported similar phenomenon. Digitization performance that excelled in smaller surface angles suddenly dropped once the angle was raised to 60°. These speculations were verified in the present study as well; prominent imprecisions were detected in scanning proximal areas above and below the contact area when evaluated from vertical and transverse cross-sections. In distal areas of proximal surfaces, scanning was especially difficult because scanner wand could not construct appropriate angle against the target surface while approaching from the anterior. 

A study published by Flügge et al. [12] concluded that imprecision in digital impression is dominated by tooth shape. In the molar areas, virtual images reproduced were more unreliable. Areas that failed to accurately reproduce may be caused by complex geometry of molars, with many angled surfaces, and undercuts of the adjacent teeth. In the present study, there was also a varying degree of deviation among the single-tooth casts. In transverse cross-sections, it was seen that teeth with deeper curves displayed the inferior precision than smoothly round teeth even though identical scanning protocols were applied.

In the present study, directionality of deviations was indicated. Interestingly, obvious curvatures in tooth geometry and protruding margins in interproximal areas commonly showed positive deviation whereas undercut areas showed negative deviation. Although individual differences exist, positive deviations could most easily be observed at mesial/distal corners in transverse-high cross-sections, and negative deviations could easily be observed in mid-transverse areas from mesial-distal cross-sections. Rudolph et al. yielded similar conclusions in his study. Molar areas with sharp angular changes were more prone to negative deviation while steep areas including mesial/distal corners showed strong positive deviation. However, some discordant results were presented in analysis of canine. Present study could not verify such results since the present study focused on posterior teeth. Rudolph et al. concluded that tooth shape was as decisive as type of scanner in data acquisition [13]. Therefore, different strategies may be considered depending on the tooth geometry despite identical procedures. 

This study was performed extraorally using stone casts. Extraoral digital impressions usually perform with higher precision than intraoral ones. Had this study tested for accuracy intraorally, results may have displayed greater fluctuations even with identical scanning protocols due to patient factors such as saliva and tongue movements. It can easily be presumed that areas that exhibited high imprecisions due to lack of space will show higher deviations in limited intraoral space [12]. Therefore, careful approach of the scanners is needed especially in interdental areas. 

Most intraoral scanners struggle to achieve acceptable level of accuracy in proximal areas. According to scanning protocols, a certain amount of image data must fundamentally be acquired for an acceptable depiction of a tooth. However, it is difficult to acquire adequate amount of images in these regions because optimal angle of the scanner is constrained by intraoral space and surrounding anatomy. The simplest way to overcome the spatial restriction is to scan the region multiple times with different angular views. Repetition with varying angle tilts can contribute more measured points in regions with strong curvature or rough angles, yielding more information. Increased constructive data will generate a more precise virtual model independent of inherent auto-correction by scan software. Meticulous depiction of the proximal corners will essentially lead to an overall increase in quality of design. Furthermore, increased image data in the otherwise troublesome surfaces would make following mid-treatment modifications on virtual model more effective, yielding improved integrity of the final prosthetic rehabilitation [9].

Conventional impression method is not perfect. Although errors are inevitable, compensation to an acceptable level during other production process results in a satiable prosthetic rehabilitation. Error may also arise during digital dental impression. However, the difference is that errors during digital impression is not compensated enough. Rather, errors in digital workflow have a cumulative effect. Internal fit and marginal gaps are common problems of dental restorations fabricated through digital production process [6]. For this reason, clinicians are inevitably more sensitive to errors during digital data acquisition. Although foremost solution to this problem will be manufacture of more reliable intraoral scanners, it is up to clinicians to explore techniques to overcome visual interferences during digital scanning.

## 5. Conclusions

Limited to the boundaries of present study, following conclusions could be drawn: Two-dimensional analysis can provide more insightful information regarding errors arising from single-tooth abutment area. Appropriate approachability, depth, reflection angles leading to complete attainment of data is important of scanners is important as much as accuracy of the scanners. That is, efforts must be made to avoid dead space, especially in proximal areas where errors are frequently made. In tooth preparation for digital impression taking, it is important to extend smooth planes without sharp edges. Sharp edges result in high deviation during intraoral scanning. 

## Figures and Tables

**Table 1:** Results of 2-dimensional analysis for two intraoral scanners (Trios 3^®^, EzScan^®^) vs. Identica^®^ at 5 cross sections (BL, MD, TH, TM, TL) expressed with RMS, mean positive deviation, and mean negative deviation values. Asterisk indicates statistical significance (*p* < 0.05).

Accuracy Results (2D)	Location	Identica vs. Trios	Identica vs. Ezscan
Root Mean Square	Buccal-Lingual	0.026 ± 0.025	0.057 ± 0.014 *
	Mesial-Distal	0.083 ± 0.074	0.094 ± 0.052
	Transverse-high	0.030 ± 0.023	0.054 ± 0.022 *
	Transverse-middle	0.037 ± 0.038	0.057 ± 0.014
	Transverse-low	0.033 ± 0.025	0.055 ± 0.018 *
Mean positive deviation	Buccal-Lingual	0.018 ± 0.017	0.052 ± 0.016 *
	Mesial-Distal	0.039 ± 0.034	0.069 ± 0.043 *
	Transverse-high	0.022 ± 0.013	0.039 ± 0.032
	Transverse-middle	0.019 ± 0.010	0.041 ± 0.023 *
	Transverse-low	0.021 ± 0.011	0.044 ± 0.015 *
Mean negative deviation	Buccal-Lingual	−0.014 ± 0.008	−0.041 ± 0.013 *
	Mesial-Distal	−0.104 ± 0.144	−0.058 ± 0.024
	Transverse-high	−0.015 ± 0.008	−0.045 ± 0.022 *
	Transverse-middle	−0.022 ± 0.024	−0.029 ± 0.011
	Transverse-low	−0.018 ± 0.011	−0.030 ± 0.012 *

* *p* < 0.05, Mean ± SD.

**Table 2:** Results of 2-dimensional analysis for accuracy at BL vs. MD and TH vs. TM vs. TL for each intraoral scanner (Trios 3^®^, EzScan^®^) expressed with RMS, mean positive deviation, and mean negative deviation values.

Accuracy Results (2D)	Trios 3	EzScan
Root Mean Square	BL vs. MD (*t*-test)	
	*p* = 0.022	*p* = 0.022
	TH vs. TM, TH vs. TL, TM vs. TL (ANOVA, LSD)	
	*p* = 0.440, 0.784, 0.616	*p* = 0.498, 0.804, 0.666
Mean positive deviation	BL vs. MD (*t*-test)	
	*p* = 0.058	*p* = 0.181
	TH vs. TM, TH vs. TL, TM vs. TL (ANOVA, LSD)	
	*p* = 0.596, 0.789, 0.792	*p* = 0.811, 0.597, 0.771
Mean negative deviation	BL vs. MD (*t*-test)	
	*p* = 0.034	*p* = 0.028
	TH vs. TM, TH vs. TL, TM vs. TL (ANOVA, LSD)	
	*p* = 0.318, 0.470, 0.779	*p* = 0.103, 0.138, 0.876

## Supplementary Materials

Supplementary File 1
