# Supplementary material for: A New Approach to Accuracy Evaluation of Single-Tooth Abutment Using Two-Dimensional Analysis in Two Intraoral Scanners
Source: Int J Environ Res Public Health. 2019 Mar 20;16(6):1021. doi: 10.3390/ijerph16061021 (PMC6466129; doi:10.3390/ijerph16061021)
Supplement: Supplementary File 1 [file ijerph-16-01021-s001.pdf]

Supplemental data

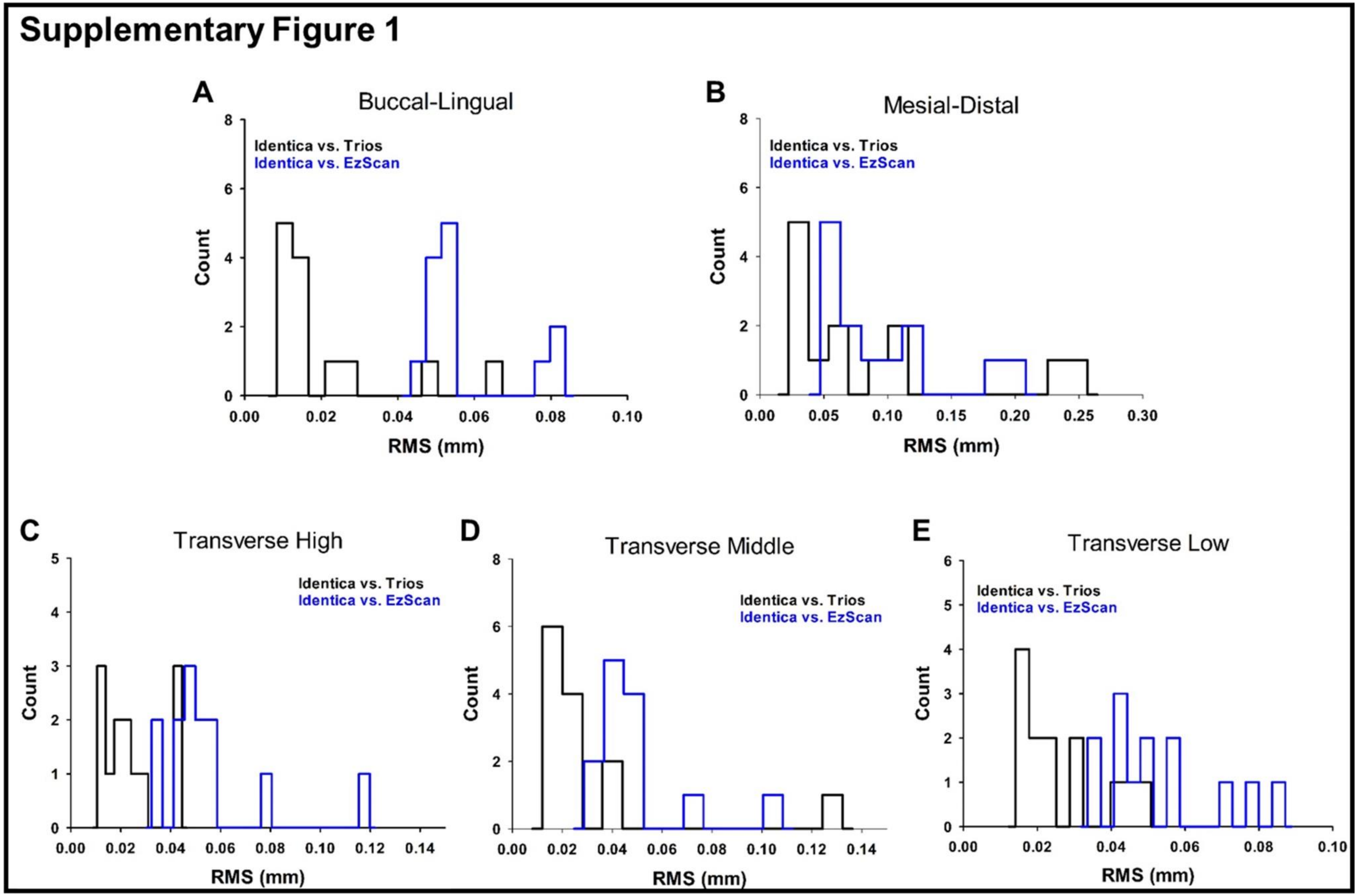

Figure S1. Histogram analysis of 2-dimensional analysis for two intraoral scanners (Trios 3®, EzScan®) vs. Identica® at 5 cross sections (BL, MD, TH, TM, TL) expressed with RMS.

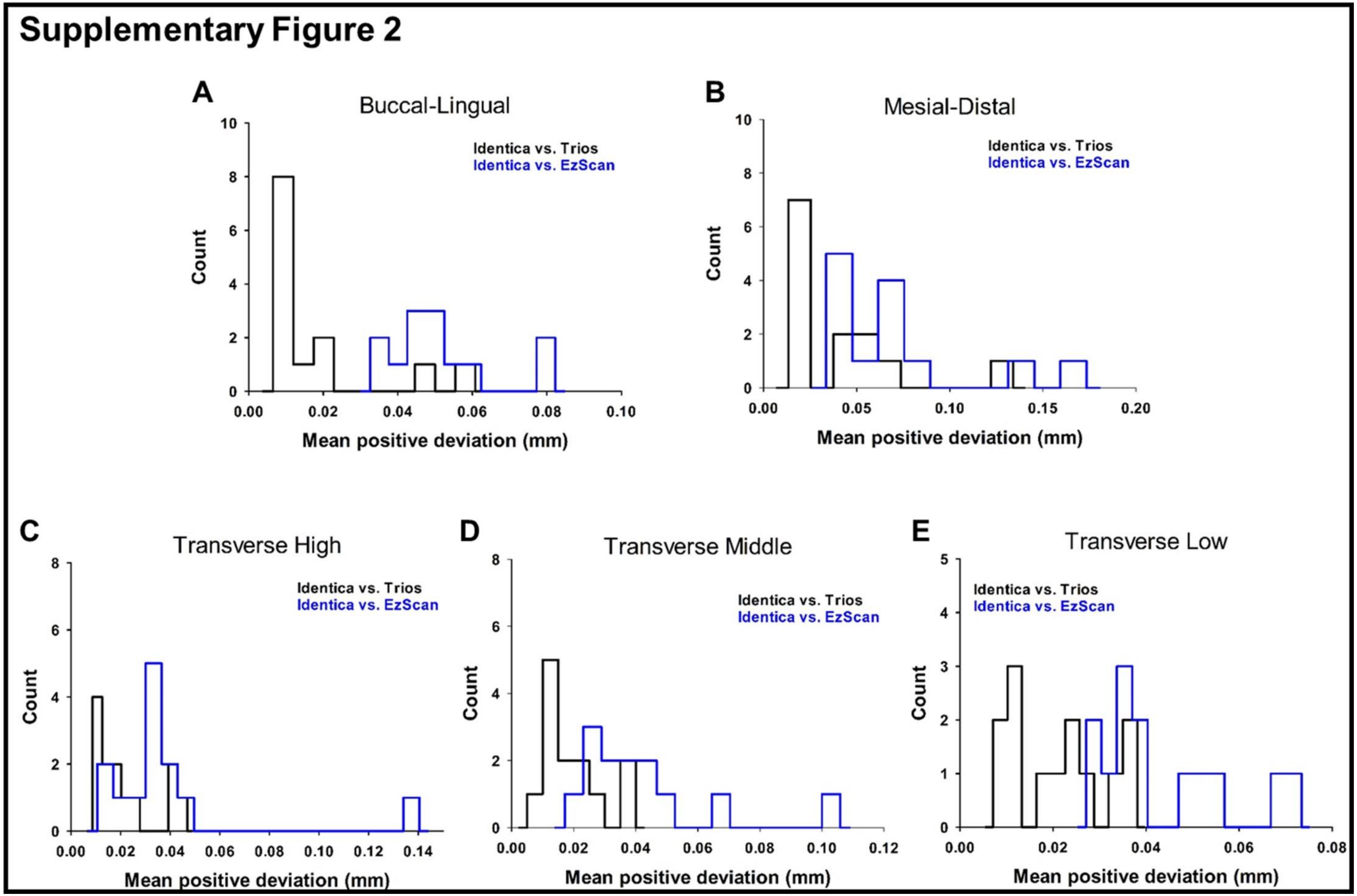

Figure S2. Histogram analysis of 2-dimensional analysis for two intraoral scanners (Trios 3®, EzScan®) vs. Identica® at 5 cross sections (BL, MD, TH, TM, TL) expressed with mean positive deviation.

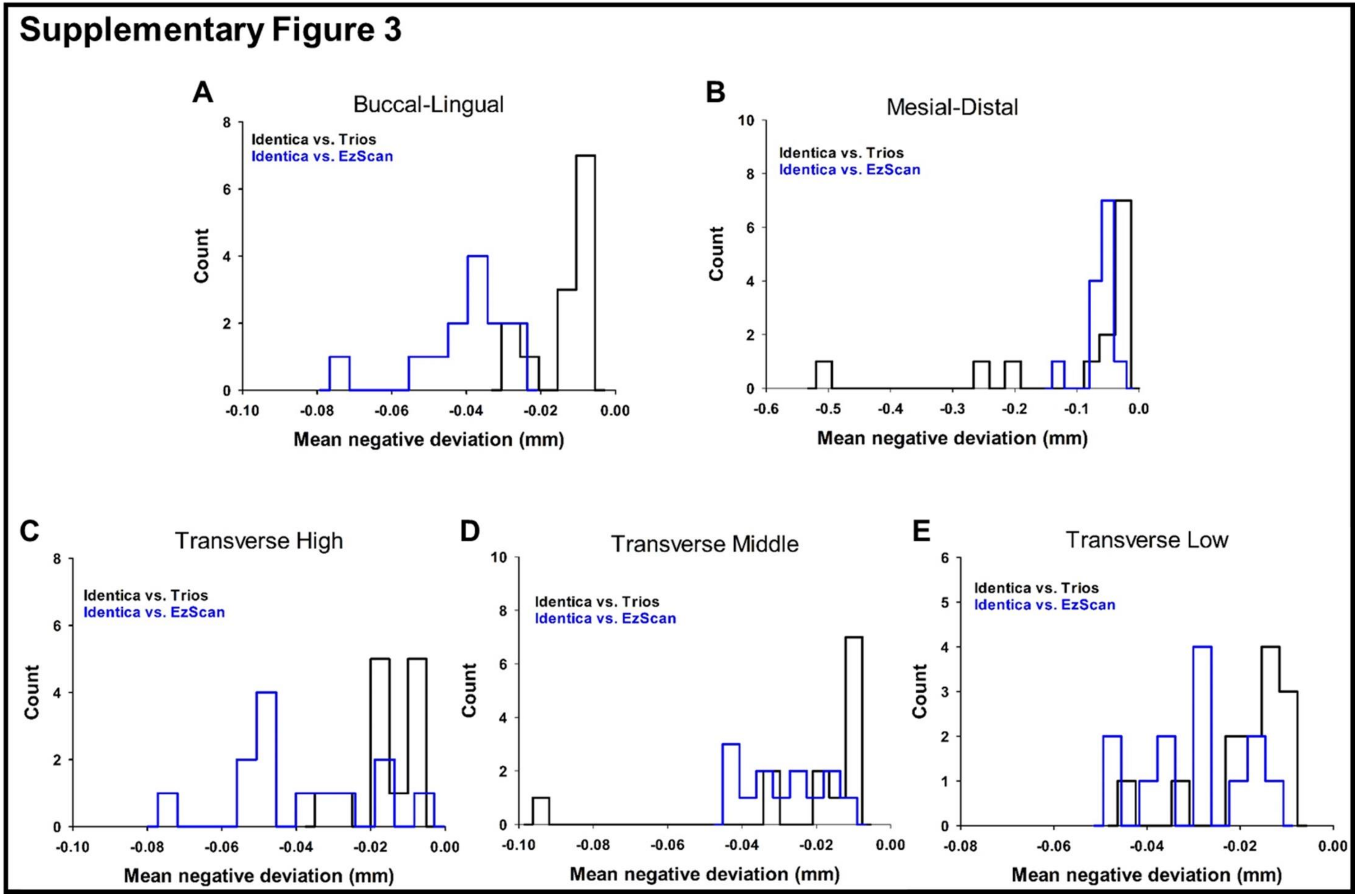

Figure S3. Histogram analysis of 2-dimensional analysis for two intraoral scanners (Trios 3®, EzScan®) vs. Identica® at 5 cross sections (BL, MD, TH, TM, TL) expressed with mean negative deviation.
